# Supplementary material for: MetaRibo-Seq measures translation in microbiomes
Source: Nat Commun. 2020 Jun 29;11:3268. doi: 10.1038/s41467-020-17081-z (PMC7324362; doi:10.1038/s41467-020-17081-z)
Supplement: Supplementary file 10 — Supplementary Data 7 [file 41467_2020_17081_MOESM10_ESM.zip › File2/Confidence_High_Taxonomy/180619_out.krona.html]

Javascript must be enabled to view this page.

members
magnitude
magnitudeUnassigned
count
unassigned
taxon
rank

180619\_out

4

superkingdom
4
2

4
phylum
1239

186801
4
class

186802
4
order

family
2
31979

genus
2
1485

2293030
2
species

SRS015794\_contig\_number\_2259SRS893270\_contig\_number\_contig-100\_324.134757

186803
family
1

genus
1
841

360807
1
species

SRS014459\_contig\_number\_42369

1897050
1
species

SRS1054716\_contig\_number\_contig-100\_36417.36418
